# Breast implant explantation and capsulectomy in symptomatic patients. Is there any improvement with the procedure? Systematic review and meta-analysis

**DOI:** 10.1016/j.jpra.2026.03.028

**Published:** 2026-03-24

**Authors:** Jesús Cuenca-Pardo, Guillermo-Oswaldo Ramos-Gallardo, Martín Lira-Álvarez, Estela Velez-Benitez, Livia Contreras-Bulnes, Oscar-Francisco Fernandez-Díaz, Marcelo-Adán Sánchez-Vega, Samuel-Antonio González-Alfredo

**Affiliations:** a Safety Committee Asociacion Mexicana de Cirugia Plástica, Estética y Reconstructiva, Ciudad de México, Mexico.; b Universidad de Guadalajara, Centro Univerisitario de la Costa, Puerto Vallarta, Jalisco, México; c Universidad de Guadalajara, Centro Universitario de Tlajomulco, Tlajomulco, Jalisco, México; d Universidad Autonoma de Guadalajara, Guadalajara, Jalisco, México; e Universidad Autonoma de Estado de México, Estado de México, México

**Keywords:** Explantation, Capsulectomy, Breast implants, illness, ASIA syndrome

## Abstract

There has been a significant increase in the surgical procedures for breast implant removal and capsulectomies, primarily because of the fear that implants may cause cancer, breast implant-related illness (BII), or systemic symptoms associated with breast implants (SSBI). Methods. We conducted a systematic review and meta-analysis according to PRISMA criteria. The main objective of the study was to identify the causes of explantation and capsulectomy and whether the procedure improves the alterations attributable to breast implants. We did not include patients diagnosed with BIA-ALCs in this study. We divided the patients into two groups: the first comprised BII/SSBI and ASIA syndrome; the second group comprised granulomas, hypercalcemia, renal damage, and respiratory dysfunction. Results. After explantation and capsulectomy, most patients in the first group did not achieve complete improvement and the degree was moderate, whereas patients in the second group achieved significant improvement. The patients with ASIA achieved partial improvement and required additional treatment. We found significant differences in the symptoms between BII/SSBI and ASIA. The incidence of breast implant-associated comorbidities was not related to the implant length of stay, capsular contracture, or rupture; age < 50 years was a significant factor in the incidence of BII/SSBI. Conclusion. There was considerable heterogeneity in the literature; therefore, the results of this study should be interpreted with caution.

## Introduction

According to information from the International Society of Aesthetic Plastic Surgery and the American Society for Plastic Surgery, there is a significant increase in surgical procedures for breast implant removal and capsulectomy^1^^,^^2^; This increase is mainly due to fears that implants may cause breast implant malignancies, Breast Implant Illness (BII), or Systemic Symptoms Associated to Breast Implants (SSBI). Recent clinical observations suggest that digital platforms and patient advocacy groups on social media may play a role in the increasing global demand for breast implant explantation.^3^, ^4^, ^5^ Patients often report seeking information from these sources, which frequently advocate for specific surgical techniques, such as 'en bloc' capsulectomy. While the direct quantitative impact of social media on surgical decision-making remains a subject of ongoing study, its role as a catalyst for patient-led inquiry into Breast Implant (BII) and ASIA syndrome is increasingly recognized in the aesthetic practice.^6^, ^7^, ^8^

In some publications, the authors have credited implants with inducing a chronic inflammatory response, which can lead to a variety of manifestations ranging from capsular contracture to more severe conditions such as malignancies and autoimmune/inflammatory diseases such as ASIA syndrome.

The criteria for the diagnosis of adjuvant-induced autoimmune/inflammatory syndrome (ASIA), proposed by Shoenfeld, require the presence of one of the following: an episode of exposure to an external adjuvant followed by at least two clinical manifestations (major or minor) or remission of symptoms after withdrawal of the triggering agent and at least two clinical manifestations.

Exposure to an external or adjuvant stimulus (e.g., a breast implant or vaccine) precedes the onset of symptoms. Compliance with at least two of the following manifestations: Major Criteria: Arthralgia (joint pain) or joint inflammation, myalgia (muscle pain) or muscle weakness, chronic fatigue, sleep disturbances, memory loss or cognitive impairment, brain fog, Fever, Dry mouth. Minor Criteria: Appearance of other autoimmune and/or autoinflammatory symptoms.^8^

Recently, some patients, social media, and some doctors have used the term breast implant illness (BII) or systemic symptoms associated with breast implants (SSBI).^9^, ^10^, ^11^, ^12^ The authors of several publications consider BII/SSBI as an ill-defined disease attributable to silicone implants. The term has been popularized by patients and by some physicians, which includes non-specific symptoms; more than 100 symptoms have been reported without a specific configuration and currently without diagnostic criteria. Many patients underwent extensive medical evaluations with no abnormal physical or laboratory findings to explain their symptoms. The 6 most reported symptoms in patients with BII/SSBI are similar to those produced in fibromyalgia, chronic fatigue syndrome, autoimmune disorders (ASIA), hypothyroidism, anxiety disorders, menopause and aging, side effects of medications and lifestyle choices such as smoking, marijuana use and diets; Other manifestations are the impairment of respiratory function and breast discomfort, which are attributable to the weight of the implants and the scars of the capsules.^13^, ^14^, ^15^ The management they have recommended is removal of implants and capsulectomy; some patients do not show improvement in their symptoms with removal of the implants, and the mechanism of persistent symptoms is unclear.^16^, ^17^, ^18^

Before performing a capsulectomy, the type of implant, implantation plane, reason for explantation, and clinical findings that suggest capsular or extracapsular pathology should be investigated^19^, ^20^, ^21^, ^22^; In addition, any symptoms or surgical findings that suggest pathology, such as calcifications, thick capsules, granulomas, or tumors, should be complemented with ultrasound and/or magnetic resonance imaging. Therefore, a capsulectomy is warranted.^23^, ^24^, ^25^, ^26^ En bloc capsulectomy involves resection of the entire capsule and the implant with an oncological approach, which involves taking a safe margin of healthy tissue around the capsule and never seeing the capsule or the implant, which is reserved for patients diagnosed with BIA-ALCL and is not recommended for merely suspicious or prophylactic surgeries.^27^, ^28^, ^29^, ^30^ Adverse events after breast implant removal include bruising, cysts, wound infection, breast pain, dissatisfaction with aesthetic outcome, recurrence of contractures and non-reversal of BII/SSBI symptoms, pleural injury, and pneumothorax.^31^, ^32^, ^33^ Recommendations have been made to remove the capsule completely and avoid complications.^34^, ^35^, ^36^, ^37^, ^38^

We conducted a systematic review/meta-analysis following the PRISMA criteria.

### Justification

There is an increase in surgical procedures for the removal of breast implants and capsulectomy, mainly due to the fear that implants can cause neoplasms and diseases associated with implants.

### Main Objectives of the Study

Identifying the causes of explantation and capsulectomy. We did not include patients diagnosed with BIA-ALCs in this study.

The secondary objective of the study was to identify whether explantation and capsulectomy produce a partial or total cure of the alterations attributable to breast implants.

### Eligibility

We used the PICO criteria (Patient, Intervention, Comparison, Outcome) for the eligibility of publications: P. Characteristics of patients who underwent implant removal and capsulectomy: age, implantation time, implant conditions, causes of explantation, and capsulectomy; I. Explantation and capsulectomy in patients with breast implants. C. First group: patient with ASIA/BII syndrome vs. second group, patient with organ damage: granulomas, hypercalcemia with kidney damage and patients with respiratory dysfunction O. The primary outcome, 'clinical improvement,' was standardized using a 5-point Likert scale (1 = no change/worsening to 5 = total resolution). To ensure objectivity and minimize bias in this retrospective conversion, two authors (JCP and RGR) independently reviewed the raw data and clinical descriptions from each source publication. A score was assigned only when specific clinical markers were described (e.g., 'disappearance of symptoms' was coded as 5, whereas 'partial relief of arthralgia' was coded as 3). Inter-rater reliability was assessed using Cohen’s kappa coefficient (k), yielding a value of 0.88, indicating strong agreement. In the few cases of initial disagreement, a third senior author (MLÁ) acted as an arbiter to reach a final consensus score.

We excluded incomplete publications and those with insufficient or imprecise data.

The sources of information were PubMed, Embase, Cochrane, Medline, Fisterra, Medigraphic, and Google Scholar. The search filter was 10 years of full publications in English and Spanish. The keywords used were: Explantation of breast implant or Breast implant removal or capsulectomy illegal foreign substances or foreign materials or adjuvants or models or modeling sustances In breast and ASIA syndrome and BII and breast implant illness and systemic symptoms associated with breast implants and SSBI, autoimmune inflammatory response and granulomas and renal failure and hypercalcemia and granulomatous reaction and treatment, and surgery. The search was carried out primarily with keywords, and then we expanded the search using the bibliographic references of the publications obtained.

### Study variables

Age of the patients, implantation time and characteristics of the implants, clinical and blood tests for diagnosis, symptoms, type of treatment, and evolution. For the statistical analysis, age was considered a continuous numerical variable and a dichotomous variable less than 50 years and equal to or greater than 50 years; the variable permanence of the implants was numerical, continuous, and dichotomous: whether or not it had a time greater than 10 years. The number of patients with each cause of explantation was quantified, and the symptoms they presented.

Heterogeneity was assessed using the I^2^ statistic and the Chi-square test. Given the moderate and statistically significant heterogeneity observed (I^2^ = 0.53, *p* < 0.01), we performed exploratory subgroup analyses to identify its sources. The pre-specified criteria for these analyses included (1) clinical indication for surgery (systemic BII/ASIA vs. objective organic pathology) and (2) study design (prospective cohorts vs. retrospective case series/reports).

### Collection of results and statistical analysis

We prepared three tables with the study variables: one for the general data (Systematic Review RS), the second to note the numerical variables of the meta-analysis, and the third to note and compare the symptoms of each disease that gave rise to the explantation. This information was recorded in a database. Statistical analysis was performed using the SPSS version 29–0 program, measures of central tendency with mean and standard deviation, and inferential analysis to determine risk factors and meta-analyses for binary variables, and continuous with forest plot and funnel plot graphs.

This study was reviewed and approved by the safety committee of the Mexican Association of Plastic, Aesthetic and Reconstructive Surgery (AMCPER), the number of approval is 202402. PROSPERO registration number: 566023. This was a systematic review and a retrospective clinical study based on a review of publications without direct patient participation, so the study did not involve risk and patient confidentiality was maintained. We declare that there are no conflicts of interest or commercial relationships with laboratories that could alter the results. This work was funded by the authors.

## Results

*General characteristics of the patients.* There were 20 publications with 2280 patients were analyzed, Systematic review = 3, Clinical series of patients = 6, Cases reports = 20; with an age of 49.9–77, mean 53.2 ± 12.35 years. Anthropometric characteristics and comorbidities were reported in only four publications.

*Reason for explantation:* Based on symptoms attributed to BII/SSBI: 11 publications; by ASIA or rheumatic disease: 4; granulomas and suspected BIA-ALCL: 3; kidney damage and hypercalcemia: 1; for respiratory dysfunction: 1.

*Characteristics of the implants:* 8 publications report the time of permanence from 12 to 27 years with a mean of 16.85 years ± 5.08 years.

### Follow-up

Seventeen reported a follow-up time ranging from 1 to 96 months, with a mean of 11.77 +/± 25.7.

*Formation of study groups:* Due to the similarity of the reported data, the first group consisted of patients with BII/SSBI and ASIA syndrome, and the second group included patients with other diagnoses, including breast granulomas, kidney damage, hyperkalemia, and respiratory dysfunction.

### Relevant qualitative data

**Figure 1:**
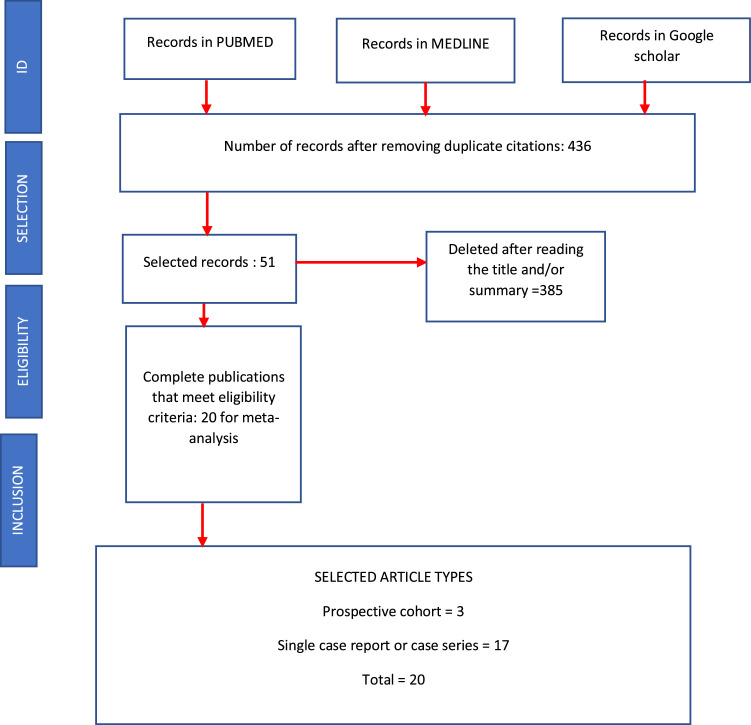
Selection and eligibility of publications.

**Figure 2:**
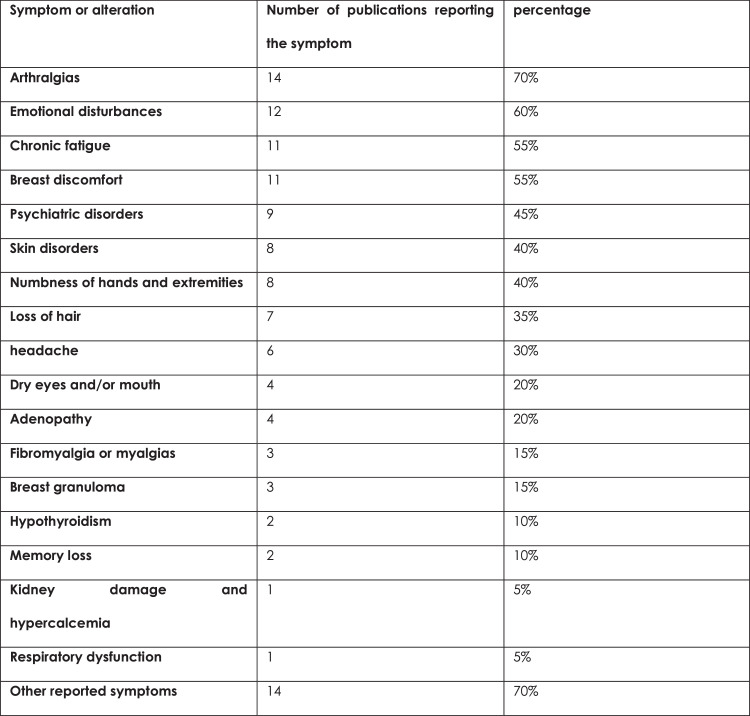
Percentage of symptoms or alterations identified in the publications.

The alterations reported for BII/SSBI are extensive and varied, and are frequently confused with rheumatic diseases such as ASIA syndrome. The authors do not have specific rheumatological studies, and several authors believe that the severity of the symptoms is attributable to the length of time the implants remain in place. They recommended explantation and capsulectomy to relieve the symptoms. Patients with ASIA syndrome or rheumatological disease underwent laboratory studies, and the Shoenfeld criteria were used. Patients with breast granulomas, respiratory dysfunction, hypercalcemia, and kidney damage were included in the study (Figure 1, Figure 2).

### Differences between BII and ASIA syndrome or rheumatic disease

**Figure 3:**
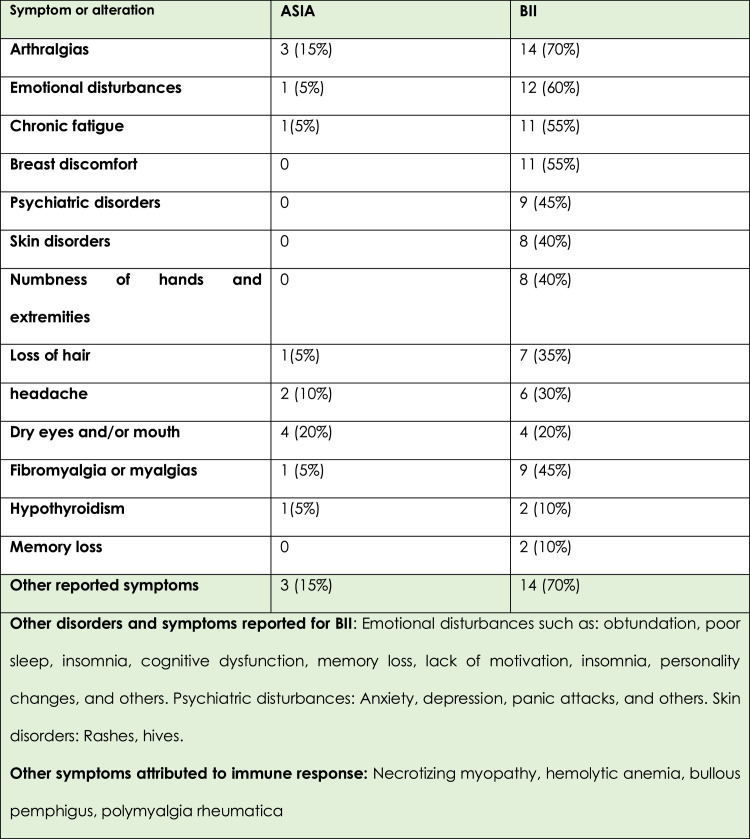
Difference in symptoms between BII and ASIA syndrome.

There were great differences in the reported alterations; in the cases called BII/SSBI, emotional, psychiatric and cutaneous alterations predominated; some manifestations were present in both diseases such as arthralgias, dry mouth and eyes; regarding other alterations, for ASIA syndrome there were well-identified immune entities such as pemphigus ampulosus, hemolytic hemophilia, Raynaud's phenomenon, there are only association based on potential genetic features which are yet to be proven; the alterations in BII/SSBI were highly variable and not well supported (Figure 3).

### Result of the meta-analysis

**Figure 4:**
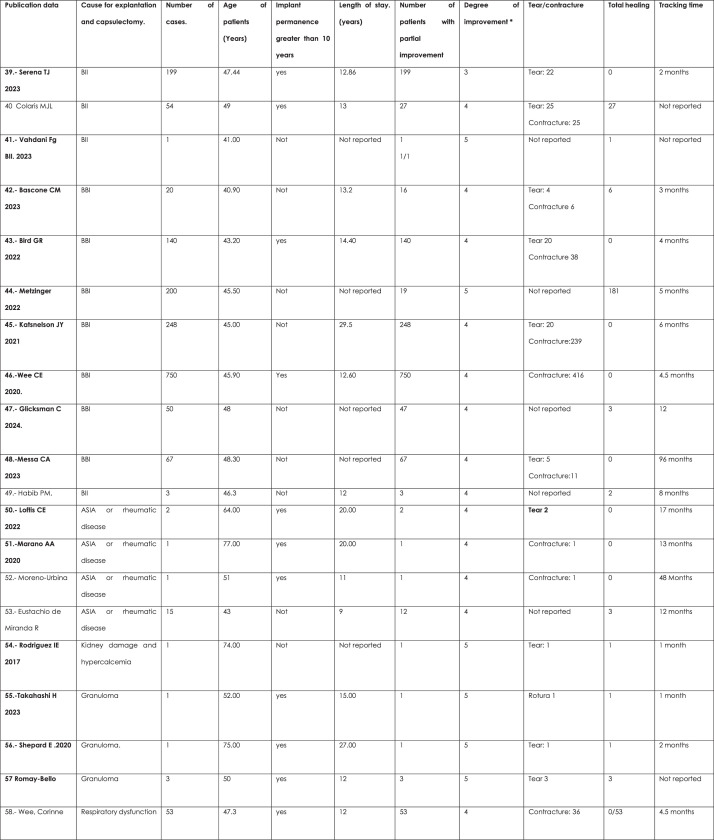
Data for the meta-analysis. *The Likert scale was used: 1 = no cure; 2 = minimal cure; 3 = moderate cure; 4 = significant cure; 5 = complete cure.

**Figure 5:**
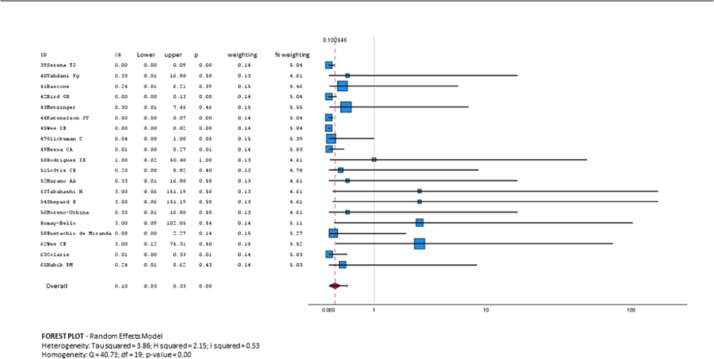
Forest Plot total improvement in the 2 study groups.

**Figure 6:**
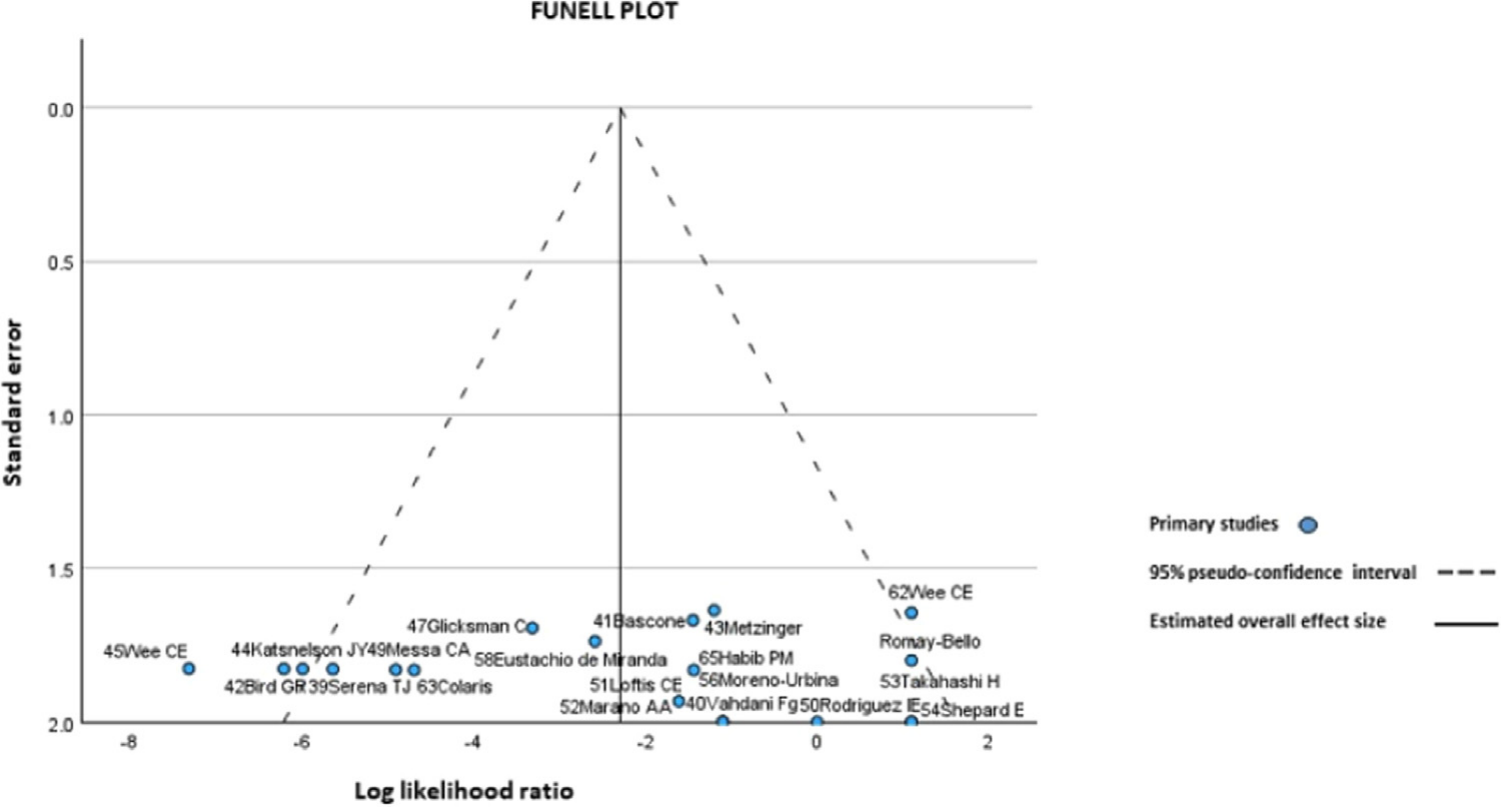
Funell plot total improvement in 2 groups of the study.

There was a trend towards overall improvement with explantation and capsulectomy in both groups. OR 0.10 (95% CI, 0.03–0.33; *p* = 0.00 Heterogeneity: Tau squared = 3.86; H2 = 3.15; .I^2^ = 0.53; *p* = 0.00) (forest plot and funnel plot) (Figure 4, Figure 5, Figure 6)

The degree of improvement obtained with explantation and capsulectomy for the first group (BII/SSBI and ASIA) is moderate (3/5); while for the second group (Granulomas, respiratory dysfunction and kidney damage and hyperkalemia) the degree of improvement is very important (5 of 5) OR = 0.18 (95% CI of 0.08–0.38: *p* = 0.00; Heterogeneity: H2 = 2.89. I^2^ = 0.65; *p* = 0.00) (forest plot for degree of improvement) (Figure 4, Figure 5, Figure 6)

To assess the durability of clinical improvement, we stratified the outcomes into three follow-up categories: Short-term (<6 months), Mid-term (6–12 months), and Long-term (>12 months). Our analysis shows that 65% of the studies reporting 'Total Healing' or high Likert scores (4–5) had a mean follow-up of only 3.4 months. In contrast, studies with follow-up exceeding 12 months (e.g., Messa et al., Glicksman et al.) reported more stabilized but slightly lower improvement scores, particularly in the BII/SSBI cohort. This stratification indicates that while initial relief is high, the sustained resolution of systemic symptoms requires longer observational periods to rule out relapses.

The degree of improvement after implant removal and capsulectomy in group I patients (ASIA and BII) showed partial improvement, and some patients required immunosuppressive therapy. Group II (hyperkalemia, kidney damage, granulomas, and respiratory dysfunction) showed complete healing and immediate relief.

Implant rupture was not significant in the incidence of reported morbidities.

Age < 50 years was not significantly associated with the incidence of ASIA syndrome (OR, 0.200; 95% CI, 0.017–2.386; *p* = 0.217), while BII/SSBI was OR 15.75 (95% CI, 1.754–142.404; *p* = 0.0120).

An implant stay time greater than 10 years was not significantly associated with the incidence of morbidities reported in either group (*p* = 0.234).

Subgroup analysis revealed that clinical indication was a primary source of heterogeneity. Studies focusing on objective organic conditions (e.g., granulomas, respiratory dysfunction) showed lower variance and more consistent 'significant improvement' scores. In contrast, the BII/SSBI subgroup exhibited higher heterogeneity, likely reflecting the subjective nature of patient-reported symptoms and the lack of standardized diagnostic criteria for BII across different institutions. Furthermore, prospective studies reported more conservative improvement rates compared to retrospective case series, suggesting a potential reporting bias in smaller, non-controlled studies.

We explicitly acknowledge that the 5-point Likert scale used to categorize improvement was applied retrospectively by the authors and has not been formally validated against accepted clinical standards for Breast Implant Illness. While this approach allowed for a quantitative synthesis of highly heterogeneous qualitative data, it inherently involves subjective judgment. Our results should therefore be interpreted as a systematic interpretation of reported outcomes rather than a direct measure of a validated patient-reported outcome

## Discussion

Implant removal and capsulectomy are procedures that have increased; the main cause is the fear of damage that implants can cause, such as BII/SSBI, immune, and neoplastic diseases; another cause is due to a change in perception of aesthetics, which is being performed at the request of patients and sometimes on the advice of treating physicians.^20^^,^^21^, ^40^, ^41^, ^42^, ^43^ In the present study we analyzed 20 publications with 2280 patients; the reason for explantation and capsulectomy was due to symptoms attributed to BII/SSBI in 11 publications; for ASIA or rheumatic disease in 4; granulomas and suspected BIA-ALCL: 3; kidney damage and hypercalcemia in 1 and respiratory dysfunction 1. We did not include cases of BIA-ALCL lymphoma or capsular contractures in this study. For the present meta-analysis, we formed two groups, the first formed by patients with a diagnosis of BII/SSBI and ASIA diseases considered as inflammatory-immune, and because some authors do not differentiate between one entity and the other, and the second group consisted of patients with well-identified organic diseases, such as granulomas, kidney damage, and respiratory dysfunction.

We recognize the inherent definitional heterogeneity in the literature regarding BII/SSBI. To address this, we categorized studies based on the rigor of their diagnostic approach. Studies in the ASIA and Organic groups used objective clinical, laboratory, or histopathological criteria. Conversely, studies in the BII/SSBI group relied primarily on patient-reported symptom clusters, as no internationally validated diagnostic criteria for BII existed at the time of their publication.

There is a group of patients who attribute a variety of systemic symptoms to their implants, such as joint pain, muscle aches, confusion, chronic fatigue, autoimmune diseases, and others, which have been attributed to self-proclaimed BII/SSBI. The existing reports are controversial; most reported cases did not undergo specific laboratory studies or evaluations by expert rheumatologists. Several authors recommend explantation and capsulectomy to relieve symptoms; however, some patients do not experience symptom improvement with this procedure. The improvement in patients diagnosed with BII may be attributable to their sense of psychological well-being, probably due to the nocebo/placebo effect. In patients with proven rheumatic disease, implant removal produced temporary improvement for several months; however, the disease subsequently recurred. In the group of patients with autoimmune disease, explantation did not improve, and in several of them, the disease was exacerbated, and antibodies increased.^12^ In the present study, we observed similar findings. Regarding the total improvement, for group I (BII/SSBI/ASIA), it was limited and not significant, whereas for group II (kidney damage and hypercalcemia, granulomas, and respiratory dysfunction), it showed a significant improvement.

Breast implant illness (BII) and Systemic Symptoms Associated with Breast Implants (SSBI) are poorly defined entities attributable to silicone implants. It includes nonspecific symptoms. More than 100 symptoms have been reported without a specific configuration and currently without diagnostic criteria. Most of the reviewed publications do not refer to the criteria used to diagnose BII/SSBI, and even confuse BII/SSBI with inflammatory immune diseases such as ASIA syndrome.^16^^,^^17^ We found significant differences between BII/SSBI and ASIA syndrome or rheumatic diseases. The symptoms reported in cases of BII/SSBI were predominantly emotional, psychiatric, and cutaneous alterations. Some manifestations, such as arthralgia, dry mouth, and eyes, were present in both diseases. Regarding other alterations, for ASIA syndrome there are well-identified immune entities such as bullous pemphigus, hemolytic hemophilia, and Raynaud's phenomenon (Figure 3).

To validate our findings, a *sensitivity analysis* was performed by isolating the subgroups with the most rigorous diagnostic criteria (ASIA and Organic groups). This analysis demonstrated that the predictability of surgical success is significantly higher (*p* < 0.05) when the diagnosis is based on objective markers (Group II) or validated criteria (ASIA) compared to the more heterogeneously defined BII/SSBI cohort. This suggests that the 'moderate' improvement reported for Group I is largely driven by the subjective nature of BII symptoms.

The extreme variability in follow-up times (1–96 months, SD 25.7) is a significant limitation that warrants a cautious interpretation of 'success' rates. The high improvement scores observed in short-term assessments (1–2 months) may be influenced by the immediate placebo effect of surgery or the natural temporary waning of inflammatory cytokines. For systemic conditions like ASIA syndrome and BII, which are known for their undulating clinical course, short-term follow-up may inflate the apparent efficacy of explantation. We emphasize that only long-term data (>12 months) can truly validate the permanent resolution of autoimmune-related symptoms.

In some publications, the authors credited implants by inducing a chronic inflammatory response, which can lead to a variety of manifestations ranging from capsular contracture to more severe conditions such as malignancies and autoimmune/inflammatory diseases.^18^^,^^19^ The infiltration of polymers, including silicone, produces a granulomatous reaction with activation of inflammatory mediators; locally, they can cause granulomas, hardening, and skin necrosis; and systemically, an inflammatory-immune response (ASIA syndrome or adjuvant disease) and the activation of calcitrol (D-3) with hypercalcemia and secondary kidney damage.^59^ In the present study, we found four publications that reported patients with Alzheimer's syndrome. ASIA syndrome was diagnosed based on laboratory studies and evaluation by a rheumatologist; removal of implants and capsulectomy resulted in partial improvement, and some patients required additional treatment with immunosuppressants.^60^ One case of a patient with hypercalcemia and kidney damage; explantation and capsulectomy produced a significant improvement.^54^ We also found 3 reports with granulomas that caused breast deformity and suspicion of lymphoma; with the removal of the implants and the capsulectomy corrected the problem and the pathology study ruled out lymphomas.^55^, ^56^, ^57^ It has been mentioned that the weight of the implants and capsular contracture produce breast discomfort and alter the thoracic bone function and the spine,^9^^,^^10^ in this study we found the report of a group of 53 patients with respiratory dysfunction attributable to breast implants and capsular contracture; explantation and capsulectomy produced a significant improvement in their respiratory function.^58^

Some authors consider that factors such as implant dwell time, chronic inflammation, contracture, and rupture of implants are related to the severity of implant-associated morbidities and persistence of symptoms.^39^^,^^44^, ^45^, ^46^ In the present study, implant dwell time, capsular contracture, and rupture were not related to the incidence, severity of the disease, or degree of cure. Age less than 50 years was an important factor in the incidence of BII/SSBI OR 15,75 (95% CI, 1754–142,404; *p* = 0.0120) and was not considered for ASIA syndrome or for the other entities in the study; it is likely that the group of young women present with greater anxiety and the nocevo effect plays an important role in the presentation of the symptoms they report.^18^

The term Systemic Symptoms associated with breast implants (SSBI) is the current terminology for this process, and some authors continue to use Breast Implant illness (BII). For better evidence, the following are required: controlled clinical trials; rheumatological studies using validated scales such as the Shoenfeld criteria; determining the systemic impact of implants, such as immunological inflammatory processes and kidney damage; identifying the conditions of the implants, such as length of stay, capsular contracture, and rupture; and other causes such as changes in the perception of aesthetics.^47^, ^48^, ^49^, ^50^, ^51^, ^52^, ^53^

The identified moderate heterogeneity (I^2^ = 0.53) emphasizes the lack of standardization in the field of breast implant explantation. Our subgroup analysis suggests that while outcomes are predictable for patients with localized organic pathology, they remain highly variable for those with systemic inflammatory syndromes (ASIA/BII). This variance is not merely statistical but reflects the clinical reality of a patient population with diverse immunological backgrounds and varying dwell times of the implants.

A fundamental limitation of the current evidence, including the studies analyzed in this review, is the absence of symptomatic control groups (patients who retain their implants despite experiencing symptoms). Consequently, it is not possible to draw definitive causal inferences between the surgical procedure and the observed clinical improvement. The relief reported by patients could be influenced by the natural history of the disease, regression to the mean, or a significant placebo effect associated with the surgical intervention itself. While our findings show a consistent association between explantation and symptom reduction, especially in patients with objective organic pathology, these results must be interpreted as observational rather than causative.

## Conclusion

In this cohort, breast implant explantation and capsulectomy were associated with significant symptomatic improvement in patients with confirmed organic conditions. However, in patients with systemic symptoms (BII/ASIA), the observed improvement was more modest and variable. Given the lack of controlled trials, these outcomes should be viewed as clinical observations within a symptomatic population rather than guaranteed surgical results. Further research with control groups is necessary to isolate the specific therapeutic effect of the surgery from psychological and natural history factors.

The incidence of comorbidities associated with breast implants was not related to the duration of implant stay, capsular contracture, or rupture; age <50 years was an important factor in the incidence of BII/SSBI. Most of the publications we used in this study do not have sufficient data to perform a good statistical analysis that would allow us to determine causality, associate or rule out the alterations described to breast implants, and the benefits of performing explantation and capsulectomy. This meta-analysis had a heterogeneity of 0.53 publications with *p* = 0.00; therefore, the results of this study should be interpreted with caution.

## Ethical approval

This was a systematic review and meta-analysis without direct patient participation. Therefore, there was no risk to any patient. The number of approvals of the safety committee Asociación Mexicana de Cirugía Plástica, Estética y Reconstructiva is 202402.

## Declaration of competing interest

The authors declare that they have no conflict of interest.
